# Nitric Oxide as a Central Molecule in Hypertension: Focus on the Vasorelaxant Activity of New Nitric Oxide Donors

**DOI:** 10.3390/biology10101041

**Published:** 2021-10-14

**Authors:** Gabriela Maria da Silva, Mirelly Cunha da Silva, Déborah Victória Gomes Nascimento, Ellen Mayara Lima Silva, Fabíola Furtado Fialho Gouvêa, Luiz Gonzaga de França Lopes, Alice Valença Araújo, Kelli Nogueira Ferraz Pereira, Thyago Moreira de Queiroz

**Affiliations:** 1 Laboratory of Nutrition, Physical Activity and Phenotypic Plasticity, Federal University of Pernambuco, Vitória de Santo Antão 55.608-680, PE, Brazil; gabriela.mariasilva@ufpe.br (G.M.d.S.); mirelly.cunha@ufpe.br (M.C.d.S.); deborah.gnascimento@ufpe.br (D.V.G.N.); ellen.mayarasilva@ufpe.br (E.M.L.S.); alice.araujo@ufpe.br (A.V.A.); kelli.pereira@ufpe.br (K.N.F.P.); 2 School of Technical Health, Health Sciences Center, Federal University of Paraíba, João Pessoa 58.051-900, PB, Brazil; fabiola.fialho@academico.ufpb.br; 3 Laboratory of Bioinorganic Chemistry, Department of Organic and Inorganic Chemistry, Federal University of Ceará, Fortaleza 60.020-181, CE, Brazil; lopeslu@dqoi.ufc.br

**Keywords:** nitric oxide, vasodilation, oxidative stress, endothelium, NO donors

## Abstract

**Simple Summary:**

Nitric oxide is an important molecule that performs a variety of functions in our bodies, especially in the cardiovascular system. In certain pathological conditions, such as cardiovascular diseases, including hypertension, there is reduced production or bioavailability of nitric oxide. Therefore, compounds that deliver nitric oxide, called nitric oxide donors, are clinically useful. In this review, we discuss the physiological role of nitric oxide, and some of the nitric oxide donors and their clinical uses, focusing on the cardiovascular system. Despite the high number of nitric oxide donors and their known efficacy, it is important to understand the similarities and differences among them and how each of them works, as well as to investigate the development of new molecules that may be better than the NO donors in current use.

**Abstract:**

Cardiovascular diseases include all types of disorders related to the heart or blood vessels. High blood pressure is an important risk factor for cardiac complications and pathological disorders. An increase in circulating angiotensin-II is a potent stimulus for the expression of reactive oxygen species and pro-inflammatory cytokines that activate oxidative stress, perpetuating a deleterious effect in hypertension. Studies demonstrate the capacity of NO to prevent platelet or leukocyte activation and adhesion and inhibition of proliferation, as well as to modulate inflammatory or anti-inflammatory reactions and migration of vascular smooth muscle cells. However, in conditions of low availability of NO, such as during hypertension, these processes are impaired. Currently, there is great interest in the development of compounds capable of releasing NO in a modulated and stable way. Accordingly, compounds containing metal ions coupled to NO are being investigated and are widely recognized as having great relevance in the treatment of different diseases. Therefore, the exogenous administration of NO is an attractive and pharmacological alternative in the study and treatment of hypertension. The present review summarizes the role of nitric oxide in hypertension, focusing on the role of new NO donors, particularly the metal-based drugs and their protagonist activity in vascular function.

## 1. Introduction

Cardiovascular diseases (CVD) are the leading cause of death worldwide [1,2]. Hypertension remains the central risk factor for cardiovascular diseases [3], and a decrease in blood pressure (BP) induces a drop in cardiovascular risk [4]. However, the treatment for high BP is complex due to the multiple mechanisms involved in the pathogenesis of hypertension [5]. It has been shown that endocrine factors, and neural and vascular reflexes, contribute to the development of hypertension and induce an increase in vascular tone [6].

Regulation of vascular tone in the vascular smooth muscle cell (VSMC) is determined by the balance between vasoconstrictor and vasorelaxant factors [7]. Among the relaxing factors derived from the endothelium, nitric oxide (NO) stands out, as it has an important role in several pathophysiological processes, such as neurotransmission, BP control, and inhibition of platelet aggregation [8]. In addition, vascular endothelium has an important protective function against cardiovascular diseases, presenting a central function in this protection [9]. In the current review, we discuss the role of NO in hypertension, highlighting the importance of NO in the regulation of the vascular response and the use of the metal-based drugs that release NO. 

## 2. Hypertension and Endothelial Dysfunction

CVD includes all types of disorders related to the heart or blood vessels. Among them, hypertension is one of the most important risk factors for heart complications and is responsible for high rates of morbidity, mortality, and hospitalization, at high cost [10]. Thus, hypertension is considered a serious public-health problem worldwide [11,12].

Hypertension is often associated with metabolic disorders, as well as functional and/or structural changes in target organs, and is exacerbated by the presence of other risk factors, such as dyslipidemia, obesity, glucose intolerance, and diabetes mellitus [13]. However, many factors are associated with these disorders and contribute directly or indirectly to the development of hypertension, including age, heredity, sex, ethnicity, social habits, stress, and others [14].

The prevalence of hypertension is increasing worldwide [2]. In 2015, 24.1% of men and 20.1% of women were hypertensive and the number of cases increased from 594 million to 1.13 billion between 1975 and 2015 [15]. It is estimated that 29% of the adult population worldwide—around 1.56 billion individuals—will have hypertension by 2025 [16]. The prevalence is 33.1% in Nigeria [17], 19.9% in Nepal [18], 30.6% in France [19], and between 13.5 and 32.5% in Brazil [20,21,22]. In the United States, the prevalence of hypertension in adults reached 46% in 2017 [23]. 

An important factor of BP control is vascular tone, which is directly influenced by the vascular endothelium. Through its multiple functions, the endothelium maintains the homeostasis of the micro-environment since it is responsible for the production of potent vasoactive mediators [24]. The endothelium is a single layer of flat polygonal cells lining the inside of all blood vessels, including arteries, capillaries, veins, and chambers of the heart. It acts as a protective layer between intra- and extravascular compartments, enabling interaction with cells and blood components [25,26]. 

Functions mediated by endothelial cells include maintenance of blood flow, regulation of inflammation and the immune response, neovascularization, and regulation of the vascular tone of the VSMC [27]. The VSMC may be regulated by endothelial cells which produce vasoconstrictor factors that promote their effects by increasing the concentration of intracellular calcium ([Ca^2+^]_i_), enhancing the sensitivity of Ca^2+^ contractile elements, and allowing Ca^2+^ influx from the extracellular fluid. Among the contractile factors released by the endothelium, we can highlight the thromboxane (TXA_2_), reactive oxygen species (ROS) such as superoxide anion (O_2_^•−^), endothelin-1 (ET-1), and angiotensin II (Ang-II) [28]. Vasodilator factors, meanwhile, have the opposite effect on [Ca^2+^]_i_ in the VSMCs [7]. The three main relaxing factors derived from the endothelium are prostacyclin (PGI_2_), endothelium-derived hyperpolarizing factor (EDHF), and NO. Endogenous production of NO is believed to be the main factor released by the endothelium for the control of vascular tone [8,28,29]. Furthermore, the vascular endothelium performs a key role in providing protection against cardiovascular diseases [9]. Damage to the endothelium generates an inflammatory response involving many types of cells (lymphocytes, monocytes, platelets, and smooth muscle), leading to dysfunction of the endothelial cells, impairment of the vascular wall, and development of an atherosclerosis plaque [30]. 

Endothelial dysfunction (ED) is characterized mainly by a reduction in the ability of endothelial cells to release NO, as determined by oxidative stress, the adhesion of leukocytes, the inflammatory response, platelet activation, and thrombosis [31]. Various other factors also induce ED, including: (1) uncoupling of the nitric oxide synthase (NOS); (2) formation of reactive nitrogen and oxygen species, reducing the bioavailability of NO and leading to nitration, nitrosylation, and oxidation of proteins; (3) oxidation or degradation of the α and β subunits of soluble guanylyl cyclase (sGC), which is the primary mediator of the bioactivity of NO; (4) greater bioavailability of vasoconstrictor agents, such as ET-1 and Ang-II; (5) oxidation of low-density lipoprotein (LDL), inducing the formation of foam cells; (6) greater expression of adhesion molecules, and; (7) increased platelet activity [32]. 

Therefore, ED is a marker for cardiovascular diseases and is shown in several pathological conditions, including diabetes, arterial and pulmonary hypertension, hyperglycemia, arthritis, obesity, heart failure, and erectile dysfunction [33]. These alterations may lead to changes in vascular hemodynamics, resulting in loss of endothelial integrity, barrier dysfunction, and atypical vasodilator and vasoconstrictor regulation, thereby modifying the vascular environment, triggering cardiovascular events, and, subsequently, increasing mortality [34].

## 3. Biosynthesis and Action of Nitric Oxide

NO is a simple small gaseous molecule which has been found to be a ubiquitous biological mediator involved in several physiological processes and plays a key role in the nervous and cardiovascular systems [35]. The molecule, previously known as endothelium-derived relaxing factor (EDRF), was first identified as NO in 1980 [36]. It has been recognized to be a signaling molecule, derived from the vascular endothelium, responsible for dilation of the blood vessels [37,38].

Since NO was pointed out as a signaling molecule in vascular relaxation, investigations have been conducted concerning the role of this gas in various biological systems in humans. NO spreads throughout the membrane and can exist in a variety of forms, such as nitroxyl anion (NO^–^), nitrosonium (NO^+^), or free radical (NO·), depending on the source of the NO [39,40].

NO is highly reactive and its relatively short half-life means that it is responsible for mediating many processes, such as endothelium-dependent vasorelaxation, platelet adhesion and aggregation, relaxation of the corpus cavernosum of the human penis, and regulation of baseline BP [41,42,43]. It also modulates inflammatory or anti-inflammatory reactions that help to regulate the numerous processes of immunological and cardiovascular systems [44,45]. 

Biosynthesis of the NO molecule occurs by the oxidation of L-arginine catalyzed by NOS. There are three known isoforms of NOS: neuronal NOS (nNOS or NOS-I) is expressed in the cytoplasm of neurons and other cell types; endothelial NOS (eNOS or NOS-III) is present mainly in endothelial cells; nNOS and eNOS are constitutive isoforms. The third isoform is the inducible NOS (iNOS or NOS-II), which is mainly associated with macrophages, but has been isolated from various other tissues, such as smooth muscle, hepatocytes, chondrocytes, microglial cells, and endothelial cells, among others [46,47,48,49,50,51,52,53]. The iNOS-produced NO is responsible for augmented leucocyte cytotoxicity against tumoral cells, bacteria, and parasites during inflammation [54].

All NOS isoforms use L-arginine as substrate and produce citrulline as a co-product. Molecular oxygen is required for the reaction and the co-factors include reduced nicotinamide adenine dinucleotide phosphate (NADPH), flavin adenine dinucleotide (FAD), flavin mononucleotide (FMN), and 6R-5,6,7,8-tetrahydrobiopterin (BH_4_) [55]. The homodimeric form of NOS is promoted/stabilized by the heme group, L-arginine, and BH_4_ [44,56,57]. However, when BH_4_ is deficient due to oxidative inactivation, the dimer of NOS breaks down, generating ROS (especially O_2_^•−^) instead of NO [58,59,60,61]. This state is referred to as eNOS uncoupling and also occurs downstream of NADPH activation, which induces oxidative stress and consequent BH_4_ deficiency and eNOS uncoupling [59,61]. Furthermore, the enhancement in ROS production can interact with NO and produce peroxynitrite (ONOO^−^), decreasing the NO bioavailability and cell damage [30,62].

Despite the similarities, there are key differences between the constitutive and inducible NOS. Firstly, the constitutive NOS activity depends on the cell calcium transient, while the basal calcium concentration is enough to maintain the activity of iNOS. Binding of the calcium–calmodulin complex is required for constitutive NOS (although its function may also be regulated by post-transcriptional events) [50,63,64], while iNOS presents the calcium–calmodulin permanently bound and its expression is mainly transcriptional controlled [65,66]. This leads to a transitory activation and low NO production by the constitutive isoforms, in contrast to long-lasting activation and high NO production by iNOS [67,68,69,70]. In addition, iNOS does not have the self-inhibition segment of the ligation site of calmodulin [71].

The expression of the inducible isoform is regulated by the induction of the synthesis of various cytokines, including interleukin 1 (IL-1), interferon-γ (IFN-γ), and tumor necrosis factor α (TNF-α) [72]. Lipopolysaccharide (LPS), an abundant molecule present in the cell wall from Gram-negative bacteria, induces cytokine production and, thus, iNOS expression [73,74]. In regular conditions, its expression is common in macrophages, where there is active inflammation, such as in alveolar macrophages in the inflamed regions of the lung [75].

**Figure 1:**
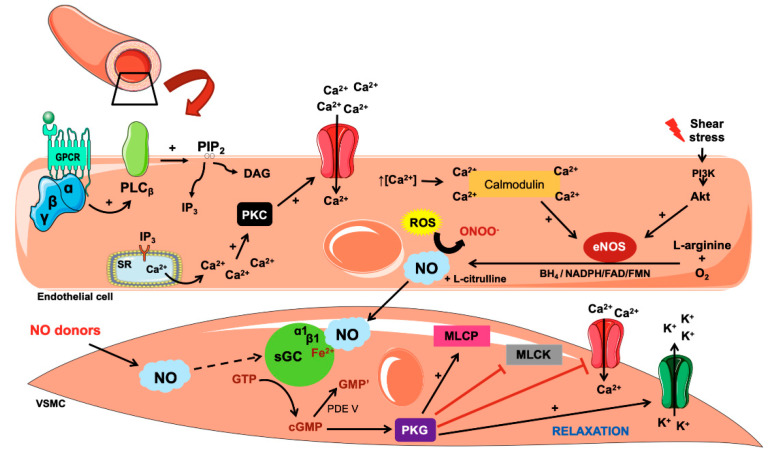
Nitric oxide (NO) induces relaxation in vascular smooth muscle cells (VSMC). The activation of G-protein coupled receptor (GPCR) stimulates phospholipase C (PLC), which is responsible for cleavage of membrane phospholipids to diacylglycerol (DAG) and inositol 1,4,5-trisphosphate (IP_3_). The latter binds to the IP_3_ receptor in sarcoplasmic reticulum (SR) to promote Ca^2+^ extrusion, which, together with DAG, evokes Ca^2+^ influx through voltage-operated Ca^2+^ channels (Ca_v_) at the cellular membrane. The linkage of Ca^2+^ to calmodulin promotes endothelial nitric oxide synthase (eNOS) activation, which, in turn, triggers the formation of NO and citrulline from arginine and O_2_. This enzyme requires cofactors such as nicotinamide adenine dinucleotide phosphate (NADPH), flavin adenine dinucleotide (FAD), flavin mononucleotide (FMN), and tetrahydrobiopterin (BH_4_). In case of an increase in reactive oxygen species (ROS), they react with NO and induce peroxynitrite (ONOO^−^) production. NO spreads to VSMC where it binds to soluble guanylyl cyclase (sGC) and causes the formation of 3,5-cyclic guanosine monophosphate (cGMP), which stimulates the cGMP-protein kinase G (PKG). This kinase negatively regulates the Ca_v_ and myosin light chain kinase (MLCK) and activates potassium channels and the myosin light chain phosphatase (MLCP). Altogether, these effects promote the relaxation of VSMC. In addition, NO can be produced from a GPCR-independent mechanism. The shear stress promotes activation of the PI_3_K/AKT pathway, which stimulates eNOS activation and subsequent NO production.

The vascular relaxation stimulus coming from the endothelium begins after vasodilator agents bind to membrane receptors in the endothelial cell or from shear stress (via PI_3_K/AKT-dependent eNOS phosphorylation) on the vascular endothelium. Once the membrane G-protein coupled receptors (GPCR) are activated by the binding of agonists, the phospholipase C (PLC) enzyme is activated, inducing a rise in diacylglycerol (DAG) inositol 1,4,5-trisphosphate (IP_3_) production. IP_3_ acts on receptors expressed in the cytoplasmic reticulum, stimulating the release of Ca^2+^ to the cytoplasm. The increase in the concentration of Ca^2+^ in the cytoplasm activates calmodulin, which, in turn, activates eNOS, which is the predominant isoform in endothelial cells. Once activated, eNOS synthesizes the NO [76,77] (Figure 1).

NO possesses the peculiar characteristic of having high affinity for heme and other iron–sulfur groups, being able to react directly with oxygen, the superoxide radical, or transition metals, such as iron, cobalt, manganese, and copper. This property is of great importance for the activation of sGC [78,79].

As shown in Figure 1, NO spreads throughout the endothelial cell, moving easily through the neighboring cells and regulating various cardiovascular effects. It crosses the endothelial space into the vascular smooth muscle, directly activating the sGC, which is the primary mediator of the bioactivity of NO, representing the largest target in muscle cells [80]. The sGC enzyme is a heterodimer, consisting of two homologous subunits, α (α1 and α2) and β (β1 and β2). The term NO-sensitive sGC has come to be used, since, apart from activating sGC, NO can also activate one of the dimers of GC (α2β1), which is found in the synaptic membrane [80].

The α1β1 dimer is the predominant isoform in most tissues, including VSMC [80,81]. In the β subunit of the dimer, the iron of the heme group binds to histidine. Once NO binds to the iron of GC, the bond with the histidine is broken. This is considered the factor that triggers the increase in enzymatic activity of sGC [80,82]. Furthermore, the activation of sGC leads to the formation of intracellular 3,5-cyclic guanosine monophosphate (cGMP), which in turn activates the cGMP-protein kinase G (PKG) pathway [83,84,85]. PKG can phosphorylate voltage-dependent Ca^2+^ channels present in the cell membrane, which causes a reduction in the entry of Ca^2+^ into the cell, thereby altering the [Ca^2+^]_i_ dynamics and constrictor function [86,87]. 

PKG uses several mechanisms to reduce mobilization of Ca^2+^ through phosphorylation and inhibition of IP_3_ formation and inhibition of the sarcoplasmic reticulum (SR)-IP_3_ receptor. NO also causes an increase in Ca^2+^ transport through the (SR) Ca^2+^-ATPase, in a cGMP-independent mechanism [88]. Furthermore, PKG acts in phosphorylate potassium channels in the cell membrane, triggering an increase in transport of K^+^ and consequent membrane hyperpolarization, thereby contributing to muscle relaxation [88]. Other ways in which PKG leads to smooth-muscle relaxation are through desensitization of the contractile filaments, inhibition of myosin light chain kinase (MLCK), and activation of myosin light chain phosphatase (MLCP) [89]. All of these effects lead to a reduced concentration of free Ca^2+^ in the cytoplasm and thus contribute to muscle relaxation (Figure 1). Moreover, PKG induces phosphorylation of vasoconstrictor targets such as TXA_2_ receptors, leading to a reduction in receptor activation and facilitating the vasorelaxant response [90]. 

Bolotina et al. (1994) found that NO also produces vasodilation through sGC-independent pathways. These mechanisms include activation of the large-conductance Ca^2+^-sensitive K^+^ channel (BK_Ca_); activation of Na^+^/K^+^ ATPase; negative modulation of Ca^2+^ channels, and; reduced sensitivity to vasoconstrictors [91]. An interesting study showed that NO produces an increase in BK_Ca_ activation by triggering rapid anterograde trafficking of BK_Ca_β1 subunit-containing endosomes in a PKG/PKA-dependent pathway [92,93]. Alternatively, cGMP may also directly activate potassium channels [94].

## 4. Mechanisms Involved in NO-Related Hypertension

The balance between the levels of NO and Ang-II seems to be a central aspect in CVD, especially in the pathogenesis of hypertension [95]. Ang-II is the most potent vasoconstrictor of the renin-angiotensin system (RAS) [96]. The effects of Ang-II are mediated by its binding to angiotensin type 1 (AT_1_R) and type 2 (AT_2_R) receptors, which are G protein-coupled receptors that induce contrary effects [97]. AT_1_R is responsible for the classic pro-hypertensive activity of Ang-II, while AT_2_R is described to present antagonistic activities compared to AT_1_R [98]. It has been shown that Ang-II (mainly by binding to AT_1_R) directly induces endothelial dysfunction and increases endothelial oxidative stress through the formation of ROS derived from NADPH oxidase [99]. Furthermore, stimulation of AT_1_R was noted as causing inhibition of eNOS, principally by phosphorylation of an inhibitory residue Tyr657 [100,101]. However, exogenous administration of an NO donor stimulates β-arrestin, which leads to desensitization of AT_1_R through its internalization, antagonizing the Ang-II effects [102]. Likewise, another study has demonstrated that NO directly interacts with AT_1_R, promoting its inhibition [95,103]. Furthermore, the addition of the exogenous NO precursor upregulated the eNOS/NO/cGMP pathway and decreased the Ang-II concentration in rats with left ventricular hypertrophy [104].

On the other hand, studies have revealed that Ang-(1–7) treatment reduced ROS formation due to a decrease in NADPH expression in the aorta of mice [105]. Moreover, evidence has shown that Ang-(1–7) induces Mas receptor (MasR) activation, a G protein-coupled receptor which stimulates the PI3K/Akt pathway, leading to phosphorylation of eNOS and subsequent NO production and release [106]. Similarly, Ang-(1–7) is able to promote AT_2_R endothelial activation, which stimulates the bradykinin (BK)–NO cascade [107,108,109]. BK is a component of the kallikrein–kinin system which acts as a counter regulator of the vasopressor RAS. BK acts through the B2 receptor and induces a decrease in ROS and NO production, enabling a reduction in BP [110].

Beyond the effect as an endothelial-dependent vasorelaxing factor, NO is also present in the brain and this gas acts as an intracellular signaling molecule and is involved in the modulation of sympathetic outflow and changes in BP [111]. Studies demonstrated that overexpression of eNOS in the nucleus of the solitary tract (NTS) or rostral ventrolateral medulla (RVLM) caused hypotension and bradycardia associated with sympathoinhibition in vivo [112,113]. In addition, recent findings have shown that NO derived from nNOS in the hypothalamic paraventricular nucleus (PVN) plays a central role in suppressing both ongoing renal sympathetic activity and BP in awake rats [114,115]. However, NOS inhibition induced neurogenic hypertension [116]. Conversely, iNOS overexpression causes hypertension with sympathetic activation due to, probably, an inflammatory condition and an increase in ROS [117]. In addition, an elegant study observed that NO originated from eNOS can alter noradrenaline (NE) release from the sympathetic nerve, inhibiting the NE release in neural/vascular tissues, and decreasing the sympathetic tone [95,118].

The first evidence of an association between Ang-(1–7) and NO in the brain was from the discovery of a co-localization of the peptide with NOS in neurons of the PVN [119]. The overexpression of angiotensin converting enzyme type 2 (ACE2), the enzyme responsible for converting Ang-II into Ang-(1–7), in the PVN, stabilized the reduction in nNOS protein expression in the PVN in animals with chronic heart failure and was accompanied by improved sympathetic nerve activity, suggesting the participation of NO in the inhibitory effects of ACE2 in the sympathoexcitation [120,121]. 

**Figure 2:**
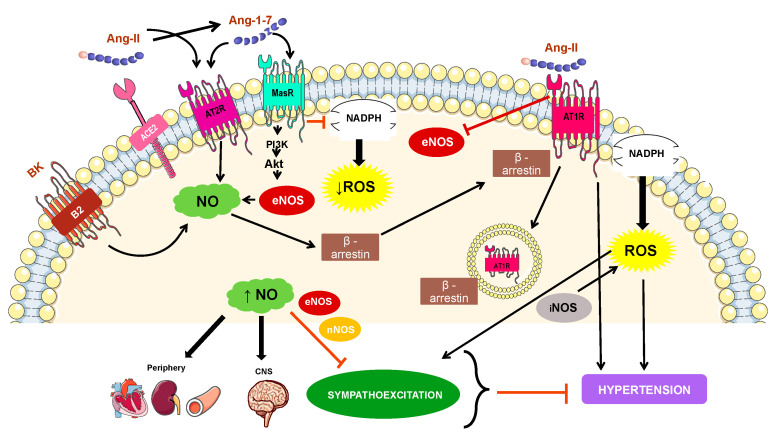
Mechanisms involved in NO-related hypertension. The effects of angiotensin II (Ang-II) are mediated by its binding to angiotensin type 1 (AT_1_R) and type 2 (AT_2_R) receptors. AT_1_R is responsible for the classic pro-hypertensive activity of Ang-II, including NADPH oxidase activation and reactive oxygen species (ROS) production. Ang-II undergoes the action of angiotensin converting enzyme type 2 (ACE2) into angiotensin 1–7 (Ang-(1–7)), which classically interacts with Mas receptor (MasR) and induces the PI3K/Akt pathway activation and consequent phosphorylation of eNOS. The activation of MasR also downregulates NADPH activity, reducing ROS levels. Furthermore, Ang-(1–7) can also bind to AT2R, which stimulates the bradykinin (BK)–NO cascade. It is important to highlight that, when activated, the BK targets such as the B2 receptors induce NO production. NO, in turn, is responsible for stimulating the β-arrestin pathway that promotes AT1R desensitization and internalization of the receptor. In summary, the increase in NO (from endothelial and neuronal nitric oxide synthase—eNOS and nNOS, respectively) promotes a decrease in oxidative stress, inducing downregulation of sympathoexcitation and consequent inhibition of BP increase in both periphery and different areas of the central nervous system (CNS). Conversely, NO derived from inducible NOS (iNOS) is involved in the rise in sympathetic tonus and ROS production, mechanisms related to triggering hypertension.

As shown in Figure 2, all this evidence together suggests that an increase in NO, especially from eNOS and nNOS sources, in the periphery, including vascular, renal, and cardiac tissues, as well as in different regions of the central nervous system (CNS) such as the PVN, NTS, and RVLM, leads to activation of the sCG/PKG pathway and a decrease in oxidative stress, inducing downregulation of the sympathetic drive and consequent inhibition of a BP increase. Furthermore, the routes that involve these effects are related to non-classic RAS (ACE2/Ang-1–7/MasR/AT_2_R) cascade overexpression and Ang-II/AT_1_R/ROS pathway decline. 

## 5. Nitric Oxide Donors

The reduced synthesis and/or bioavailability of NO are associated with many CVDs, including arterial hypertension, atherosclerosis, coronary diseases, and angina [122]. Regarding the properties of NO, a large number of NO donor compounds have emerged as potential agents for the treatment of the aforementioned diseases, able to exploit the wide variety of biological functions. Thus, pharmacological aspects of NO are constantly under study [45,123,124,125,126,127]. Furthermore, administration of drugs that mimic the effect of NO on the organism is an attractive proposal, since this is a pharmacological alternative that could reverse and/or prevent cardiovascular disorders [125].

The pathways for the formation and consequent release of NO differ significantly depending on the class of compounds and their reactivity [123]. The amount of NO released by a donor is one important factor, since cardiovascular action only occurs at very low concentrations and higher concentrations are toxic [128]. Some NO donor compounds require a catalytic enzyme to release NO, while others release NO spontaneously, without an enzyme. On the other hand, other NO donors need an interaction with thiol groups, some being reduced, and others oxidized, but all depend on the exposure time [123,129].

Prospection of nitrosylated compounds has emerged as a possible source for the formation of NO-releasing agents in biological targets, which could induce the relaxation of the vascular smooth muscles. Due to the endothelial dysfunction that occurs in some pathologies, NO donors have been developed to overcome the deficiency in this molecule, although some tolerance to organic nitrates has been reported [130].

### 5.1. Sodium Nitroprusside (SNP)

One of the best-known NO donors is sodium nitroprusside (SNP), which presents a short half-life and high reactivity with oxygen [38]. SNP is an inorganic complex used, since 1928, as a vasodilator in hypertensive crisis and cardiovascular emergencies, such as angina pectoris and heart failure [131,132,133]. SNP also provides a controlled hypotensive effect during surgery [134]. Furthermore, SNP is frequently employed as a nitrovasodilator prototype in pharmacological studies. However, provision of NO from SNP requires only light irradiation or the reduction of one electron [123,134]. 

The main clinical limitation of SNP is the release of NO accompanied by the release of cyanide (CN^−^), which forms part of its structure, making it highly toxic to the organism and causing long-term treatment to lead to endothelial dysfunction [135]. Furthermore, intravenous administration of SNP brings on a rapid, sharp drop in arterial pressure and consequent reflex tachycardia [136]. Therefore, tolerance, the formation of CN^−^, reflex tachycardia, and endothelial dysfunction are all factors that limit the use of these NO donors, in view of their undesirable side-effects.

### 5.2. Organic Nitrates

Organic nitrate NO donors are the oldest class of donors used in cardiovascular medicine [137,138]. This group of NO donors includes organic nitrate esters with a nitroxyl (-O-NO_2_) and can be used as a monotherapy or in combination with other drugs. Glyceryl trinitrate (GTN), isosorbide mononitrate (ISMN), and isosorbide dinitrate (ISDN) are the most frequently prescribed, while pentaerythrityl tetranitrate (PETN) is little recommended because it does not have proven effectiveness [139,140,141,142]. The mechanisms involved in the anti-angina effect induced by organic nitrates include reduction in the preload, which is induced by peripheral vasodilation and, in minor extension, by dilation of the epicardial coronary artery and reduction in systemic BP [143]. The effects of organic nitrates on preload and afterload lead to reduced oxygen consumption in the heart, in addition to promoting increased oxygen supply due to dilation of both non- and atherosclerotic coronary arteries [144,145].

GTN and PETN have little oral bioavailability, with approximately 90% being metabolized by the liver. However, they can be administered intradermally or sublingually. On the other hand, nitrates such as ISMN, ISDN, and nicorandil are bioavailable orally, but with a quick duration of effect [145,146].

GTN is the class prototype and the organic nitrate that has been most widely studied to date [147]. GTN is a prodrug metabolized by mitochondrial aldehyde-dehydrogenase (ALDH-2) that converts GTN into nitrated metabolite (1,2-gylceryl dinitrate) and nitrite (NO_2_). NO is a result of NO_2_ reduction or interaction between the two metabolites [148].

### 5.3. Clinical Use and Limitations of Nitric Oxide Donors

The in vivo effects of organic nitrates are well established and possess some advantages compared to other classes of nitrates [123,148,149,150]. The therapeutic benefits of nitrates are related to their effects on peripheral and coronary circulation. Clinically, the inhalation of NO has been approved for primary pulmonary hypertension in newborns [151]. In addition, GTN-induced exhaled NO has been shown to be a valuable tool to monitor metabolic function of the pulmonary vasculature, in contrast to endogenous NO in exhaled breath, which could be a marker of the production and consumption of NO in the airways [152]. Recent studies demonstrated that there are remarkable changes in GTN-induced exhaled NO after cardiopulmonary bypass (CPB). In fact, there was a significant reduction in the increase in exhaled NO induced by GTN at 1 and 3 h after CPB [153]. Thus, this finding indicates that, although NO production/consumption in the airway compartment may remain intact after cardiac surgery, consumption reaction may dominate in the microvascular compartment [154].

Another use of NO donors is in chemo- and radiotherapy. NO donors have a role in enhancing the tumor perfusion to improve tumor therapy [155]. In an interesting clinical trial, transdermal administration with GTN improved the indicators in patients with advanced cell lung cancer [156,157]. 

When administered by the oral route, organic nitrates present variable oral bioavailability, due to a variable rate of hepatic first-pass metabolism [146,158,159,160]. It is rapidly absorbed (reaching plasma in a few minutes) and distributed and is also quickly cleared from the plasma [158,159,161]. Metabolism may be through non-enzymatic and enzymatic systems [161]. On the other hand, inorganic nitrites/nitrates do not undergo first-pass metabolism, presenting, thus, high bioavailability [162,163,164].

Organic nitrates are metabolized by different pathways, which are either of an activating or degrading nature. Degrading routes for GTN yield inorganic nitrite and nitrate, and glyceryl-1,3-dinitrate. Degradation is accomplished by glutathione reductase (GR) and glutathione-S-transferase (GST) [165,166]. Bioactivation routes lead to NO, S-nitrosothiols, inorganic nitrite, and glyceryl-1,2-dinitrate [167]. For the organic nitrates in general, various pathways are described for organic nitrate bioactivation, such as cytochrome P450 superfamily (liver, but not vascular), deoxyhemoglobin, deoxymyoglobin, and xanthine oxidase, GSH-S-transferase [166,168,169]. Activation of mitochondrial aldehyde dehydrogenase (mitALDH) is predominant and this mechanism has a key role in nitrate tolerance [147]. The relative role of each enzymatic GTN biotransformation pathway in a given tissue or specialized cell type may be influenced by factors such as its prevailing abundance, isozyme pattern, and substrate specificity [124]. The major nitros(yl)ation sites for GTN are the heart and liver [124].

The inorganic nitrites/nitrates follow the nitrate–nitrite–NO activation pathway, and may be through heme proteins, deoxymyoglobin, xanthine oxidase, endothelial Nitric Oxide synthase, and aldehyde oxidase, among others [170,171,172,173,174,175]. Moreover, mammalian commensal bacteria may reduce nitrate to nitrite [176,177]. 

As elegantly reviewed by Omar and colleagues, 2012 there are some differences in the therapeutic uses of organic and inorganic nitrites/nitrates. While organic nitrates have a negative impact on endothelial function through the production of ROS [177], inorganic nitrites/nitrates present a positive impact. Both induce a fall in SBP, but the fall induced by organic nitrites/nitrates present rapid onset, while inorganic is slower. The use of organic nitrates is highly limited by the induction of tolerance, while there is no evidence of tolerance for inorganic nitrates. Both organic and inorganic nitrates lead to a reduction in pulmonary arterial pressure when inhaled [145].

A number of adverse effects of the organic nitrates are known. The acute effects, such as hypotension, dizziness, nausea, and headache, are associated with the vasodilator effect [145]. The notable effects associated with chronic use are nitrate tolerance, increased oxidative stress, and endothelial dysfunction [145,165]. Inorganic nitrites/nitrates may also induce the acute effects of reflex tachycardia [136], but, differently, there is no evidence of tolerance for inorganic nitrites/nitrates [178]. In addition, a carcinogenic effect in rodents has been observed with the use of certain nitrosamines [179].

It is already known that these compounds can be used to treat cardiovascular diseases, such as acute myocardial infarction, and hypertensive emergencies, due to their vasorelaxant properties [167,180]. However, long-term administration of organic nitrates has been shown to diminish their hemodynamic effects [181]. Long-term treatment with GTN causes tolerance and consequent loss of the hemodynamic effect [182] and also induces endothelial dysfunction [167]. In addition, clinical trials have demonstrated contradictory effects regarding their use in atherothrombotic diseases, especially with long-term nitrate use [183].

A mechanism which leads to nitrate tolerance involves increasing levels of endothelin within the vasculature, activation of PLC and protein kinase C (PKC), and a subsequent increase in actomyosin activity and myocyte contractility. Moreover, activation of the RhoA/Rho kinase pathway contributes to vasoconstriction by inhibition of MLCP [180,184,185]. In addition, continuous treatment with GTN induces NOS dysfunction, probably by reduction in BH_4_ bioavailability [186]. 

Tolerance induced by nitrates can also comprise the desensitization of sGC, resulting in decreased responsiveness to NO [184,187]. Furthermore, a remarkable study revealed that GTN metabolism induces ROS production following oxidation of thiol groups in the active site of ALDH-2, which may cause inhibition of ALDH-2 enzyme activity and reduce GTN efficacy [141,188]. In addition, another type of tolerance, called pseudo-tolerance, which is characterized by dysfunction in neurohormonal systems such as elevated catecholamine release rates and circulating catecholamine levels, sodium retention, and intravascular volume expansion, however this phenomenon is induced in response to every vasodilator therapy [141,189].

Differently from other organic nitrates, PETN does not induce tolerance in animals or humans [190,191], probably because it does not induce an increase in vascular production of ROS, as seen with GTN [192,193], and did not change ALDH-2 activity [194].

It has been shown that PETN therapy improves pulmonary hypertension beyond its known cardiac preload reducing ability [195] and may be beneficial in the treatment of ischemic heart diseases involving oxidative stress and impairment in nitric oxide bioactivity [196]. Furthermore, PETN induced a reduction in BP in SHR female but not male offspring of mothers fed with a high-fat diet. It also diminished ACE expression, profibrotic cytokines, and kidney fibrosis, suggesting epigenetic changes [197,198].

In a model of superimposed preeclampsia and high-fat diet, maternal PETN treatment showed both beneficial (improved glucose tolerance) and unfavorable effects (increase in blood pressure and decrease in EDHF-mediated vasodilation in the offspring) [199]. 

Molsidomine belongs to the group of sydnonimines. It is metabolized in the liver to SIN-1, which does not require enzymatic bioactivation, so NO is released spontaneously in the arterial wall [200,201]. In rats, it has been seen that the administration of molsidomine did not improve pathological changes in the cardiovascular system in SHR [202], but in rats with renal mass reduction, it normalized systemic blood pressure and partially ameliorated renal disease progression, with these effects being potentiated by lisinopril [203]. In combination with other drugs, molsidomine decreased cardiac fibrosis and stabilized systolic function in a model of chronic renocardiac syndrome [204]. Moreover, it attenuated the hypoxia-related effects that lead to pulmonary hypertension [205]. Perinatal administration of molsidomine increased renal vascular resistance and ameliorated hypertension and glomerular injury in adult fawn-hooded hypertensive rats, a model of mild hypertension, impaired preglomerular resistance, and progressive renal injury [206].

In humans, it has been observed that treatment with linsidomine and molsidomine was associated with modest improvement in the long-term angiographic result after angioplasty, although it had no effect on clinical outcome [207]. It also improved flow-mediated vasodilation in patients with artery disease [208], and induced antianginal effects and a decrease in the levels of ICAM-1, which is correlated with the severity of atherosclerosis [209], suggesting an important role in this pathology.

Substantial evidence has shown that NO is involved in many inflammatory conditions. Studies have demonstrated that NO can be pro-inflammatory or anti-inflammatory. Due to the dual effects promoted by NO, this phenomenon is often referred to as the NO paradox [210].

The production of ONOO^–^ is a potent oxidant agent that can deeply impair the regular functions of biological systems such as endothelial integrity. In spite of the short half-life of this oxidant at physiological pH, the interaction of ONOO^–^ with the cellular membrane and molecules with biological activity provokes damaging outcomes in pathophysiological oxidative-stress conditions [211], which include inhibition, inactivation, or activation of enzymes, modification in protein structure, and disorders in signaling pathways and cellular energetic disbalance [212]. Studies have verified that ONOO^–^ induces activation of COX-1 and COX-2 enzymes with subsequent production of prostaglandins [213]. Nevertheless, ONOO^–^ could also inhibit COX activity mediated by nitration of tyrosin385 residue, producing a divergent response in regard to prostanoid formation [214,215].

There are differences in the ways in which NO is released, the amount of NO generated, and the time during which it is released from the NO donors mentioned above. S-nitrosoGlutathione (GSNO) is found in vivo and is an important intermediary in organic nitrate metabolism. The remaining nitrosothiols are synthetic. These compounds act as intermediates in the nitrosylation of proteins and possess the ability to transfer the different NO species through chains of thiols, without releasing the NO molecule itself. This action mitigates the probability of NO reacting with O_2_^•−^, generating ONOO^–^, or reacting with other molecules to nitrosylate them [216]. Sydnonimines release NO spontaneously, without enzymatic participation. O_2_^•−^ is generated concomitantly and together with NO leads to ONOO− formation. This reaction causes production of hydroxyl radical, increasing its prooxidant potential [217]. SNP does not release NO spontaneously in vitro, but requires partial reduction (one-electron transfer) by a variety of reducing agents shown in membrane cells. In addition to NO, SNP can release, in aqueous solution, a range of oxidant and free radical species, such as iron, cyanide, superoxide, H_2_O_2_, and hydroxyl radical [218,219,220,221].

NO also acts as an anti-inflammatory through the impairment of monocyte adhesion, as well as the expression of proinflammatory target genes of NF-κB, such as TNF-α, IL-6, iNOS, ICAM, V-CAM, and COX-2 in vessels as well as in glial cells [222,223,224,225,226]. However, the trigger for these signaling pathways is based on cell type, concentration of NO donor (in vitro studies), administration route, and cell-redox state [221]. For this reason, the conclusion is that NO cannot be rigidly classified as either an anti-inflammatory or a pro-inflammatory gas [210]. 

Despite the potential of NO in medicine, only two types of NO donor drugs, SNP and organic nitrates, are currently used in the clinic [127]. Other classes of NO donors are available for clinical studies, such as the derivatives of a minor heterocycle system, the furoxan ring. 4-Methyl-3-phenylsulfonylfuroxan is one of the most active products of the furoxans. Its ability to inhibit platelet aggregation induced by arachidonic acid in human plasma is reversed by the presence of HbO_2_^++^, a well-known scavenger of NO. Furthermore, it increases cGMP levels in human platelets in a dose-dependent manner and inhibits the increase in Ca^2+^ concentration induced by arachidonic acid [227].

New chemical classes of NO donors have now been synthesized and may have potential for the treatment of CVDs. Our research group and others have studied some new NO donors which induced hypotension in normotensive rats such as (Z)-ethyl 12- nitrooxy-octadec-9-enoate (NCOE) [126] and cis-[Ru(bpy)_2_(py)(NO_2_)](PF_6_) (RuBPY) [125] as well as in hypertensive animals, for instance 2-nitrate-1,3-dibuthoxypropan (NDBP) [228,229,230], the nitrosyl–ruthenium complex [Ru(terpy)(bdq)NO^+^]3^+^ (TERPY) [231,232,233,234], the cyclohexane nitrate (HEX) [235], and the organic nitrate 1,3-bis (hexyloxy) propan-2-yl nitrate (NDHP) [236] (Table 1).

### 5.4. Metal-Based Drugs as NO Donors

Stretching back 5000 years, compounds containing metal ions have been widely used to treat various diseases [237]. As many of these compounds contain a metal in their structure and possess pharmacological properties, they are known as metal-based drugs [238].

Recently, metal-based drugs such as ruthenium compounds have been studied as NO donors and are attracting increasing interest, particularly due to their stable active forms under physiological conditions and low toxicity, thereby making them suitable for clinical use [233]. This class could provide a new source of NO-releasing agents in biological targets, especially for relaxation of VSMC. 

The use of metals for the development or modification of pharmaceutical products has numerous advantages, including a variable number of geometries and forms of coordination, accessibility to different redox states, and specific thermodynamic and kinetic characteristics, in addition to the intrinsic properties of metal cations and their ligands, which sometimes undergo significant alterations when a metal complex is formed [239,240,241]. 

The biological properties of ruthenium complexes were first reported in the 1950s [242], and prospection of nitrosyl compounds with transition metals has raised the prospect of the formation of NO-releasing agents and, in particular, redox release of NO in biological targets. Drugs capable of activating the intracellular receptor of NO, sGC, independently of endothelial NO have been developed to address the issue of tolerance produced by nitrates. 

In view of the promotion of such actions by NO and the side-effects of NO donor drugs, such as nitrate-tolerance in clinical settings, some research groups, including ours, have been studying metal-based NO donor drugs that act on the cardiovascular system, with a potential action to induce vasorelaxant activity and BP and decrease oxidative stress, among other effects [45,243,244,245,246,247,248].

The trans-[Ru(Cl)NO(cyclam)^2+^ complex has been shown to sustain a more prolonged hypotensive effect, averaging 15 min, which is almost 20 times the duration of the effects of SNP, in either normotensive or hypertensive mice [249]. Another study using a ruthenium complex demonstrated that trans-[RuCl([15]aneN_4_)NO]^2+^ induced rat aorta dilatation only in the presence of a reducing agent by the sGC-cGMP pathway and potassium-channel activation, which leads to a decrease in cytoplasmic Ca^2+^ concentration. This compound releases NO· and NO^−^ species [243,244].

A ruthenium compound that has been extensively studied, called TERPY, also induced aorta relaxation through sGC-cGMP and potassium-channel pathways, but not sarcoplasmic reticulum Ca^2+^-ATPase activation [245]. Interestingly, TERPY failed to induce vascular relaxation in rat basilar arteries, probably due to impairment in enzymatic bioactivation of the NO donor in this vascular bed [246]. This should be pointed out as an important characteristic as cerebral vasodilation is believed to be the major reason for NO donor-induced headaches [250]. On the other hand, the relaxation induced by TERPY was similar in mesenteric resistance arteries from Sham and two-kidney-one-clip hypertensive rats (2K1C), but not in aorta, probably due to impairment in the potassium-channel activation induced by TERPY in this last vessel [251,252]. Furthermore, although less potent than SNP, TERPY induced a long-lasting effect which was greater in 2K1C than in normotensive rats. This long-lasting effect could be related to the slow release of NO, which could be an interesting characteristic of TERPY as a potential therapeutic vasodilator [232]. In aortas from spontaneously hypertensive rats (SHR), the relaxation induced by TERPY was not different from Wistar control rats, and neither was the amount of NO released by the compound [233]. Moreover, the effect of TERPY was improved by the presence of endothelium and eNOS in SHR, through uncoupling and hyperphosphorylation of eNOS [253]. Additionally, the hypotensive effect of TERPY was greater in male than in female SHR, probably due to oxidative stress [254].

Another metal-based drug complexed with ruthenium, RuBPY, which has a nitrite instead of NO in its moiety, required the presence of the tissue to release NO, showing its stability in solution. The likely enzyme responsible for this release is sGC. RuBPY induced relaxation through the NO–cGC pathway in rat aortas [255]. However, in cultured VCMC from rat aorta, RuBPY was able to induce NO· release that activates K^+^ channels in an sGC-independent pathway [256]. On the other hand, RuBPY induced relaxation in rat mesenteric resistance arteries through NO–sGC–cGMP–PKG-pathway activation, but not through K^+^ channels or SERCA triggering [42]. 

Concerning hypertensive animals, Pereira et al. (2017) investigated the effect of RuBPY in different arteries in 2K1C rats. The authors observed that the relaxation was similar in aorta, mesenteric resistance, and coronary arteries between normotensive and 2K1C rats, although it was smaller in basilar arteries from 2K1C than in normotensive rats. Moreover, differently from SNP and similar to TERPY, RuBPY did not induce a hypotensive effect in normotensive rats. Altogether, these data may indicate advantages of RuBPY over SNP, since it does not induce an effect in normotensive rats, while it did induce coronary artery relaxation (which may be useful for angina) and a minor effect in the basilar artery (which may indicate that it does not induce headache) [125]. Furthermore, differently from GTN, RuBPY did not induce self-tolerance or cross-tolerance with acetylcholine, which could be another advantage of this NO donor for clinical use [257]. 

Importantly, the NO donors may have distinct characteristics. Ru(NO)(salenCO_2_H)Cl was able to induce vascular relaxation of rat aorta only in the presence of light, possibly due to photolabilization from the ruthenium nitrosyl [258]. Oishi et al. (2015) have suggested that cis-[Ru(H-dcbpy-)_2_(Cl)(NO)] (DCBPY) at low concentration (0.1 µM) is not an NO generator, but can inactivate ROS and improve endothelial function [259].

Based on promising compounds studied by us and other Brazilian colleagues, our collaborators have synthetized new metal-based drugs containing the NO molecule to induce potential beneficial effects in the cardiovascular system. Many preclinical studies have been performed to support this concept. An NO donor similar to RuBPY, called Rut-bpy (Cis-[Ru(bpy)_2_(SO_3_)(NO)]PF_6_) induced stabilization in BP in anesthetized hypotensive Wistar rats [260]. Cerqueira et al. (2008) studied two related nitrosyl–ruthenium complexes, named cis-[Ru(bpy)_2_(SO_3_)(NO)]PF-6-9 (FONO1) and trans-[Ru(NH_3_)4(caffeine)(NO)]C1_3_ (LLNO1), which demonstrated a potent vasodilator effect in rabbit corpus cavernosum [261], corroborating the vasodilator potential of these drugs.

Recently, new nitrosyl–ruthenium compounds, denominated *FOR*, have been produced from a simple and easy route and tested in the cardiovascular system and other organic systems, demonstrating remarkable outcomes. The cis-[Ru(bpy)_2_(2-MIM)(NO)](PF_6_)_3_ (FOR811A) was studied in a murine model of allergic asthma and it decreased the alveolar collapse and preserved the bronchoconstriction during asthma. In addition, molecular docking using FOR811A showed a strong interaction with the heme group of cGC [262]. Another compound, cis-[Ru(NO_2_)(bpy)_2_(5NIM)]PF_6_ complex showed a potential pharmacological application as an antioxidant and anti- inflammatory (inhibition of pro-inflammatory cytokines) in in vitro studies [263].

Finally, a very recent study demonstrated that the new ruthenium-based nitric oxide donor cis-[Ru(bpy)_2_(ImN)(NO)]^3+^ (FOR0811) administered intravenously by bolus infusion or chronically using subcutaneous implanted osmotic pumps, decreased BP, presenting a long-lasting effect, and did not demonstrate reflex tachycardia in L-N^G^-Nitro arginine methyl ester (L-NAME) hypertensive rats [248]. In addition, FOR0811 induced a reduction in the low (LF) and very low (VLF) frequency bands. The authors also detected a vasorelaxant response in aortic rings mediated by the sGC–cGMP pathway after addition of FOR0811 [248]. Furthermore, FOR0811 evoked relaxation in human corpus cavernosum and was able to increase cGMP levels, and this effect was either blocked or reversed by a cGC inhibitor, the 1-H-[1,2,4] oxadiazolo-[4,3-a]quinoxaline-1-one (ODQ) [264]. These responses elicit the new ruthenium complex as a promising NO donor to treat cardiovascular dysfunctions. At this moment our research group is concentrating on the study of metal-based drugs similar to FOR0811 (unpublished data). Notable results on vascular activity and BP have been revealed, encouraging us to continue the study of these compounds with a future perspective to their use in humans (Table 2).

## 6. Conclusions

In this paper, we briefly reviewed the role of NO in the cardiovascular system, focusing on its involvement in vasodilation. Based on the important properties of NO, NO donors were produced and have been used for the treatment of vascular disorders. However, due to the side effects presented by these donors, particularly vascular tolerance, new molecules have emerged with the potential to be used in the treatment of cardiovascular diseases. In this context, new organic nitrates such as NDBP, NCOE, and NDHP, and especially metal-based drugs with NO in their structure, have been studied by our research group and collaborators. Among them, TERPY and RuBPY have been widely explored, which demonstrated robust hypotensive and vasorelaxant responses in several models of hypertension. In addition, we are currently investigating other new ruthenium complexes named FOR. These studies have shown very promising results, which lead us to continue the analyses in an attempt to abolish the harmful effects presented by other NO donors used in clinical practice. 

## Figures and Tables

**Table 1:** Classic NO donors.

No Donor	Class	Clinical Uses	Clinical Limitations	References
Sodium Nitroprusside (SNP)	Inorganic donor	-Vasodilation in hypertensive crisis and cardiovascular emergencies, such as angina pectoris and heart failure -Hypotensive control during surgery	-Formation of CN^−^ -Reflex tachycardia -Endothelial dysfunction -Tolerance	[38,123,131,132,133,134,135,136]
Molsidomine	Sydnonimines	-Vasodilation in patients with artery disease -Antianginal effects	-Despite an improvement in the long-term angiographic result after angioplasty, it induced no effect on clinical outcome	[200,202,207,209]
Glyceryl trinitrate (GTN)	Organic nitrate	-Antianginal effect (reduction in the preload by peripheral vasodilation and dilation of the epicardial coronary artery) and reduction in systemic BP -Increase in oxygen supply due to dilation of both non- and atherosclerotic coronary arteries	-Small oral bioavailability -Endothelial dysfunction -Tolerance -Increases oxidative stress -Increases autocrine endothelin expression -Induces supersensitivity to vasoconstrictors	[143,144,145,146,167,181,182,183]
Isosorbide mononitrate (ISMN)	Organic nitrate	-Vasodilation for the treatment of angina pectoris -Vasodilation of coronary arteries	-Short effect, despite the oral bioavailability -Endothelial dysfunction -Therapy of post-infarct leads to an increased rate of coronary events -Increases oxidative stress -Increases autocrine endothelin expression; -Supersensitivity to vasoconstrictors	[143,144,145,146]
Isosorbide dinitrate (ISDN)	Organic nitrate	-Vasodilation for the treatment of angina pectoris -Vasodilation of coronary arteries	-Short effect, despite the oral bioavailability	[143,145,146]
Pentaerythrityl tetranitrate (PETN)	Organic nitrate	-Improvement in pulmonary hypertension beyond reduction in the preload -Treatment of ischemic heart diseases -Does not induce tolerance	-Little oral bioavailability	[143,144,145,146,195]
Nicorandil		-Vasodilation for chronic stable angina	-Short effect, despite the oral bioavailability	[142,143,144,145,146]

**Table 2:** New chemical classes of NO donors.

No Donor	Class	Effect	Species	Tolerance	References
(Z)-ethyl 12-nitrooxy-octadec-9-enoate (NCOE)	Organic nitrate	-Short-lasting hypotension and bradycardia -Vasorelaxation	Rat	Does not cause in vitro tolerance	[126]
2-nitrate-1,3-dibuthoxypropan (NDBP)	Organic nitrate	-Hypotension, bradycardia, and bradypnea -Prevention of the progression of angiotensin II-mediated hypertension	Rat	Does not cause in vitro tolerance	[228,229,231]
Cyclohexane Nitrate (HEX)	Organic nitrate	-Reduction in blood pressure and heart rate -Antihypertensive effect in renovascular hypertension -Vasorelaxation in cranial artery	Rat	-	[235]
1,3-bis (hexyloxy) propan-2-yl nitrate (NDHP)	Organic nitrate	- Reduction in blood pressure in hypertensive animals -Vasorelaxation -Prevention of the progression of hypertension and endothelial dysfunction	Rat	Does not cause in vitro tolerance	[236]
[Ru(terpy)(bdq)NO^+^]^3+^ (TERPY)	Metal-based drugs	-Vasorelaxation in aorta and mesenteric resistance arteries from Sham and two-kidney-one-clip hypertensive (2K1C) -Long-lasting hypotensive effect in 2K-1C, but not in normotensive -Similar vasorelaxation and released NO in aortas from Wistar and Spontaneously Hypertensive Rats (SHR) - Does not induce vasorelaxation in basilar arteries -Hypotensive effect in SHR	Rat	-	[231,232,233,234,245,251,252,254]
[Ru(bpy)2(py)(NO2)](PF6) (RuBPY)	Metal-based drugs	-Induced relaxation in aorta, mesenteric resistance arteries; coronary arteries between normotensive and 2K1C rats -Did not induce hypotensive effect in normotensive rats -Induced coronary artery relaxation (which may be useful for angina) and a minor effect in basilar artery (which may indicate that it does not induce headache). -NO· release that activates K+ channels in cultured VCMC aorta	Rat	Does not cause in vitro tolerance (self- or cross-tolerance)	[42,125,255,256,257]
trans-[Ru(Cl)NO(cyclam)^2+^	Metal-based drugs	-Long-lasting hypotensive effect (20 times greater than SNP) in normotensive and hypertensive animals	Mouse	-	[249]
trans-[RuCl([15]aneN4)NO]^2+^	Metal-based drugs	-Vasorelaxation in aorta (due to the release of NO· and NO-species)	Rat	-	[243,244]
Ru(NO)(salenCO2H)Cl	Metal-based drugs	-Vasorelaxation in aorta	Rat	-	[258]
Rut-bpy (Cis-[Ru(bpy)2(SO3)(NO)]PF6	Metal-based drugs	-Stabilization of BP in anesthetized hypotensive animals	Rat	-	[260]
cis-[Ru(bpy)2(SO3)(NO)]PF-6-9 (FONO1)	Metal-based drugs	-Vasodilation in *corpus cavernosum*	Rabbit	-	[261]
trans-[Ru(NH3)4(caffeine)(NO)]C13 (LLNO1)	Metal-based drugs	-Vasodilation in *corpus cavernosum*	Rabbit	-	[261]
cis-[Ru(bpy)2(2-MIM)(NO)](PF6)3 (FOR811A)	Metal-based drugs	-Decrease in alveolar collapse and prevention of bronchoconstriction during asthma	Mouse	-	[262]
cis-[Ru(bpy)2(ImN)(NO)]^3+^ (FOR0811)	Metal-based drugs	-Decrease in BP (long-lasting) with no reflex tachycardia in L-NG-Nitro arginine methyl ester (L-NAME) hypertensive rats -Reduction in the low (LF) and very low (VLF) frequency bands in rats -Vasorelaxation in rat aorta -Vasorelaxation of human *corpus cavernosum*	Rat and human	-	[248]

## Data Availability

Not applicable.
